# Effect of Grape Seed Extract on Gelatin-Based Edible 3D-Hydrogels for Cultured Meat Application

**DOI:** 10.3390/gels9010065

**Published:** 2023-01-12

**Authors:** Kummara Madhusudana Rao, Hyeon Jin Kim, Soyeon Won, Soon Mo Choi, Sung Soo Han

**Affiliations:** 1 School of Chemical Engineering, Yeungnam University, 280 Daehak-ro, Gyeongsan 38541, Republic of Korea; 2 Research Institute of Cell Culture, Yeungnam University, 280 Daehak-ro, Gyeongsan 38541, Republic of Korea

**Keywords:** gelatin, proanthocyanidins, hydrogel, cell growth, cell adhesion, cultured meat

## Abstract

Cell-cultured meat, which is artificial meat made by in vitro cultivation of animal-derived cells, has attracted a lot of interest as a potential source of protein in the future. Porous hydrogels are crucial components that can be used as an artificial extracellular matrix (ECM) to provide cell growth for generating cultured meat. In this study, we highlight the effects of grape seed extract (proanthocyanidins, PC) on the physicochemical and biological functions (bovine satellite muscle cell (BSC) growth and adhesion) of an edible gelatin (GL)-based hydrogel. The freeze-dried hydrogels had good compressive characteristics with pore sizes ranging from 100 to 300 μm. BSCs were able to grow and attach to porous GL-PC hydrogels. These studies suggested that the developed hydrogels using edible materials and made by employing a low-cost method may serve in the cell growth of muscle cells for cultured meat applications.

## 1. Introduction

Recent advancements in meat production technologies, such as cell-cultured meat, have the potential to partially replace the existing animal business [1]. The progress of this technology presents a potential remedy for the conflict between conventional methods of meat production and sustainable development [1,2]. The tissue engineering approach has recently been utilized by combining biomaterials, cells, and growth factors to produce meat-like analogs for cultured meat applications [3]. Scaffolding biomaterials are required in tissue engineering to produce a 3D-like microenvironment for cell growth and differentiation [4]. Hydrogel-based scaffolds for cultured meat provide a 3D gel-like microenvironment with specific biochemical and biophysical cues that are controlled by the biochemistry and mechanical characteristics of the extracellular matrix (ECM) [4].

Among the many different kinds of materials available for use in biomedical applications, the naturally occurring protein gelatin (GL), which is found in the bones, skin, and connective tissues of animals, shows particular promise and offers the following benefits [5]: the Food and Drug Administration (FDA) considers this material to be safe, biocompatible, and biodegradable. It has the arginine-glycine-aspartic acid (RGD) peptide motifs that are known to be essential in encouraging cell adhesion and are less immunogenic than collagen [6]. Improved cell adhesion encourages the growth and differentiation of bovine myoblasts [7]. Additionally, it offers a variety of functional groups for crosslinked chemical and physical changes. Aldehydes and aspartic and glutamic acids are widely used in chemical crosslinking operations, whereas heating, drying, and irradiation processes are used in physical crosslinking methods [8]. Based on these features, GL-based scaffolds, microcarriers, and fibrous scaffolds have been used for cultured meat applications [9]. Therefore, GL-based scaffolds could fulfill the structural and biochemical feature requirements of natural meat tissues.

Grapefruit seed extract, which is high in flavonoids, is an effective natural antimicrobial agent certified for safety [10]. Proanthocyanidins (PCs) are naturally occurring polyphenolic compounds which are widely found in fruits and vegetables. They are a type of condensed tannin that is composed of highly hydroxylated molecules that can form insoluble complexes with proteins and carbohydrates. According to the information that is currently available, PCs speed up the transformation of soluble collagen into insoluble collagen throughout development. Compared to glutaraldehyde, PCs are about 120 times less cytotoxic, and their crosslinked matrices promote cell ingrowth and proliferation [11].

Based on the edibility and physicochemical features of GL and PCs, we report herein the preparation of a hydrogel-based porous scaffold for cultured meat applications. The hydrogels were analyzed based on their physicochemical characteristics for culturing BSCs. The results show that the GL-PC hydrogel could have potential and be beneficial for cultured meat applications.

## 2. Results and Discussion

### 2.1. Physicochemical Features of GL-PC Porous Hydrogels

**Figure 1:**
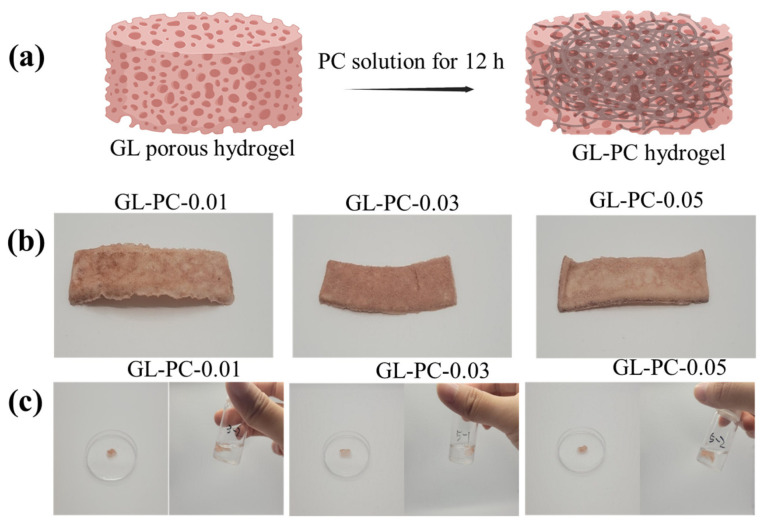
(**a**) Schematic representation of preparation of GL-PC porous hydrogels for cultivated meat production, (**b**) digital photograph images of GL-PC hydrogels after freeze-drying, and (**c**) digital photograph images of the stability of GL-PC hydrogels in PBS (pH 7.4) at 37 °C.

In this work, first the GL hydrogel was prepared by a freeze-drying method followed by immersion in various amounts of PC solutions. At this stage, most of the PC molecules easily interacted with GL via H-bonding, thereby strengthening the GL-PC hydrogel. In general, catechol-based tannins and other compounds can easily be complexed with GL [12]. Similarly, the PCs were also easily complexed with GL after the GL hydrogel was immersed in the PC solution. The complexation is mainly due to the abundant catechol groups on PCs, which easily interact with GL via H-bonding interactions. The uncrosslinked GL hydrogel easily dissolved in PBS solution at 37 °C. To confirm the stability of the GL-PC hydrogels, they were immersed in PBS solution and incubated for 24 h at 37 °C. In the digital images, the GL-PC hydrogels appeared to be swollen rather than dissolved, indicating the PCs were strongly bound to the GL via H-bonding interactions (Figure 1).

**Figure 2:**
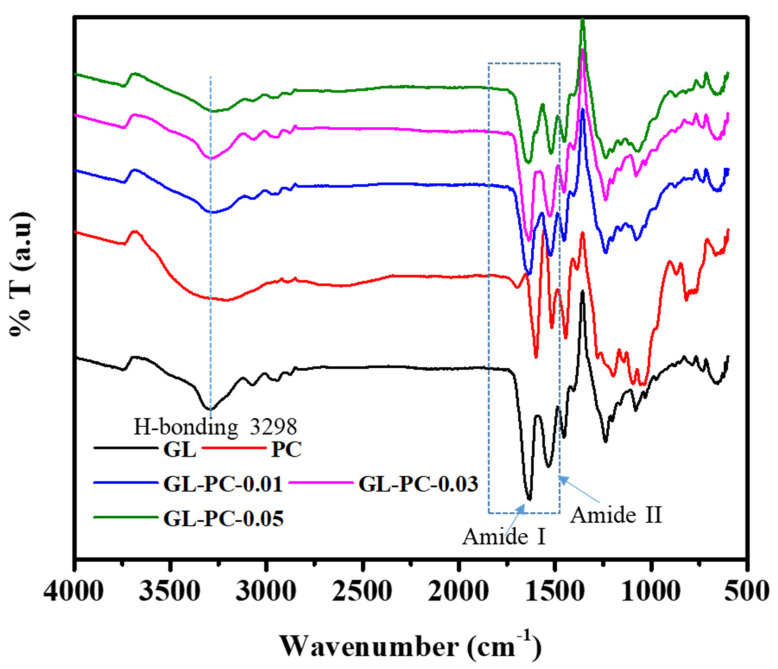
FTIR spectra of GL-PC hydrogels.

Furthermore, the freeze-dried hydrogels were characterized by FTIR spectral measurements to confirm the H-bonding interactions between GL and PC. As shown in Figure 2, GL shows peaks at 3298 cm^−1^ and 3067 cm^−1^, representing the stretching vibrations of amide I and amide II of the NH functional group, respectively. The peaks observed at 1630 cm^−1^ and 1530 cm^−1^ represent the stretching vibrations of amide I and bending vibrations of amide II (N–H), respectively. A peak appeared at 1237 cm^−1^, assigned to the C–N stretching vibration. PC shows major peaks at 3255 cm^−1^ due to phenolic –OH groups, at 1602 cm^−1^, representing aromatic C–C bonds, and 1044 cm^−1^ due to C–O stretching vibrations. The FTIR spectrum of the GL-PC-0.01 hydrogel showed an absorption peak at 1630 cm^−1^, representing the amide I (C=O stretching), which was similar to that seen in GL. The peak at 1525 cm^−1^ shows a decrease in the amide II bending value compared to pure GL, which indicates H-bonding with PC. Furthermore, the amide I (N–H) stretching vibration at 3288 cm^−1^ was broadened and strengthened compared to that in GL, which further indicates H-bonding with PC. The amide I stretching (N–H) and amide II bending (N–H) values were further decreased with increasing PC concentration, proving the H-bonding interactions formed between GL and PC.

**Figure 3:**
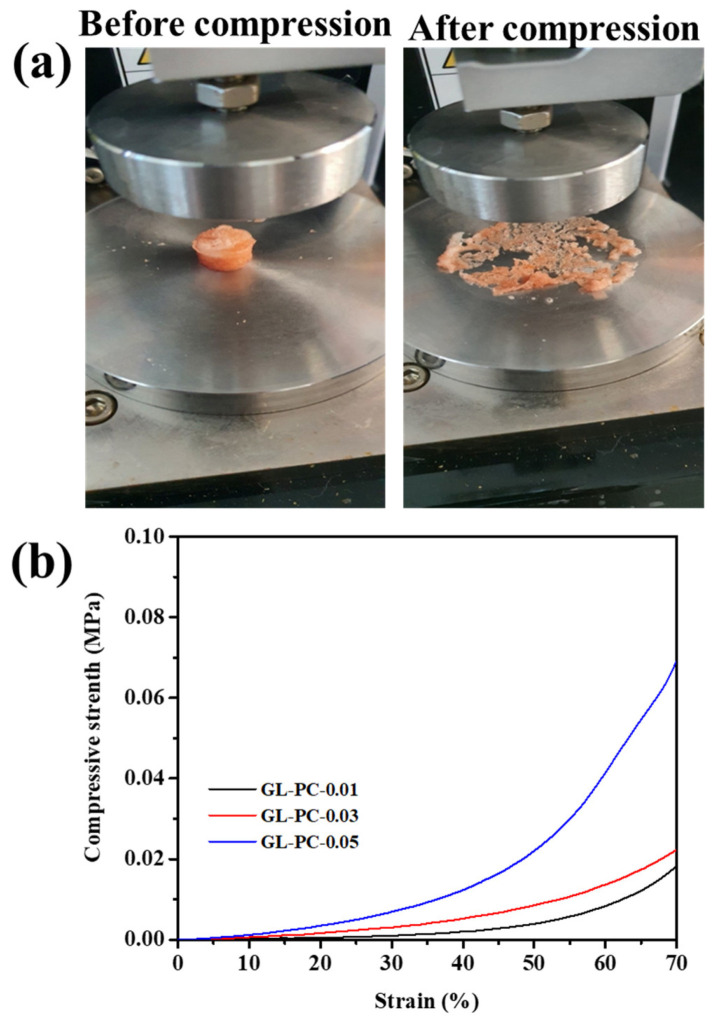
(**a**) Digital photographs of GL-PC hydrogels before and after compression and (**b**) compressive analysis of GL-PC hydrogels.

Cell viability can suffer from exposure to strong shear stress from the flowing cell growth media in bioreactors. The porous hydrogel scaffold wall architecture or a protective, flexible, and elastic surrounding gel can help scaffolding of 3D cultures decrease or regulate shear stress [4]. To further confirm the stability of GL-PC hydrogels, a compressive analysis of the hydrogels was performed. As shown in Figure 3a, the digital photographic images of the compression of the hydrogels showed that the GL-PC hydrogels did not recover after compression. The compressive strengths of GL-PC0.01, GL-PC0.03, and GL-PC0.05 were 1.16, 1.32, and 1.58 MPa, respectively (Figure 3b). The compressive strength values significantly increased with the increasing amount of PC hydrogels due to the improved complexation between the GL and PC. The compressive moduli for GL-PC0.01, GL-PC0.03, and GL-PC0.05 at 5% strain were 1.12, 1.36, and 1.41 kPa, respectively. These values were similar to those of bovine muscle at 1.2–1.8 kPa under 1–5% strain [13]. Therefore, the developed hydrogel scaffolds are more advantageous for producing meat-like tissue in bioreactors.

**Figure 4:**
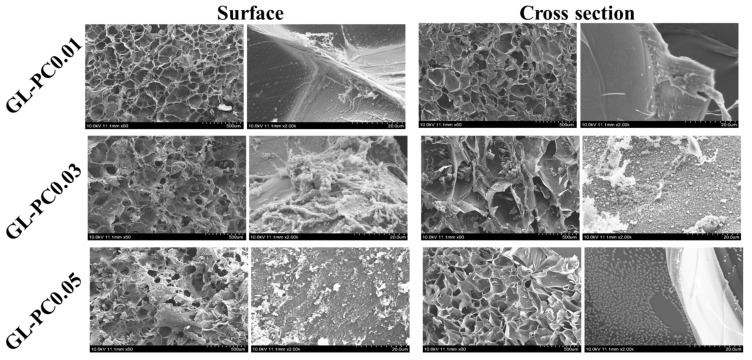
SEM images of GL-PC freeze dried hydrogels.

The sponge-like structure of the porous hydrogels with pore sizes in the range of tens to hundreds of microns provided the mechanical stability needed for the seeded cells to form tissues and deposit ECM. For muscle tissue engineering, these hydrogels should mimic the structure, mechanical characteristics, and composition of the perimysium connective tissue, given that the hydrogel should continue to be a crucial part of the mature tissue [14]. Therefore, freeze-dried hydrogel morphology is very important for muscle tissue formation. The morphology of the freeze-dried hydrogels is shown in Figure 4. The SEM images show the scaffolds are porous structures on the surface as well as in the cross-section. The hydrogel had good compressive characteristics with pore sizes ranging from 100 to 300 μm. With the increasing concentrations of PCs, they self-aggregate and interact with the GL via H-bond interactions. The self-aggregation is mainly caused by pi–pi static bonding between the catechol groups of PCs. The self-aggregation of the PC nanostructures may improve the hydrogel stability and be beneficial for cell growth.

**Figure 5:**
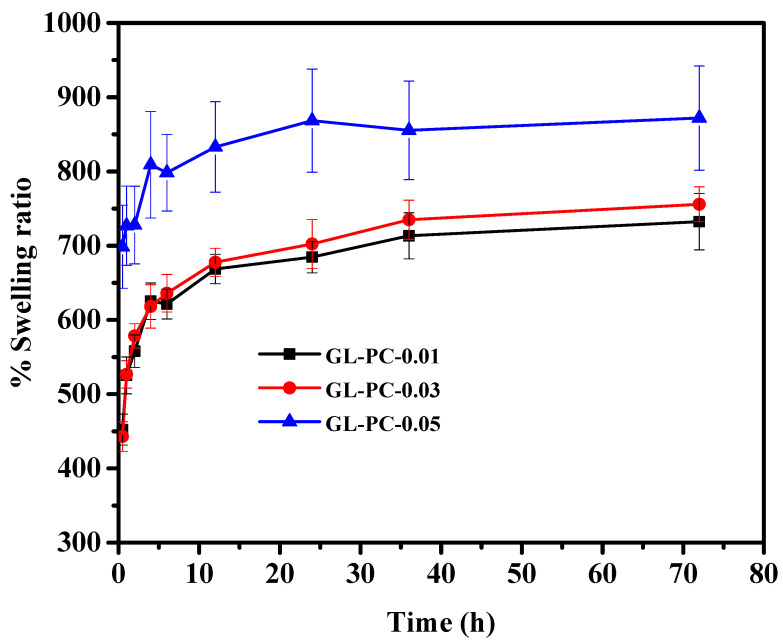
Swelling study of GL-PC hydrogels in PBS at 37 °C.

One of the primary characteristics of the biocompatibility of hydrogels is swelling, which occurs when the hydrogel matrix absorbs a significant amount of water and has characteristics similar to those of living tissues in terms of physiological stability and nutritional permeability [4]. The immersion of the freeze-dried hydrogels in PBS (pH 7.4) showed a significant water uptake within 1 h of incubation. The abundance of catechol groups in PCs, which are readily solvated in water, caused the swelling to increase as the concentration of PCs in the hydrogels increased (Figure 5). The equilibrium swelling was achieved within 70 h of incubation in the PBS solutions. The GL-PC0.05 hydrogel showed the maximum swelling capacity due to both the solvation of the hydrophilic segments and pore filling by the PBS solvent, since the hydrogels have good porosity [15,16].

### 2.2. Biological Function of Porous Hydrogels with BSCs

**Figure 6:**
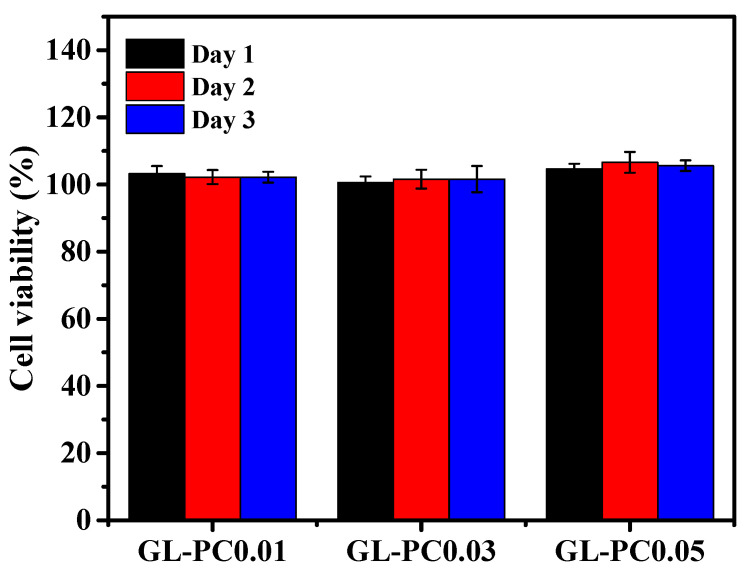
Cell viability BSCs (isolated from 30-month-old-Korean male cattle) cultured on GL-PC scaffolds using PrestoBlue assay.

**Figure 7:**
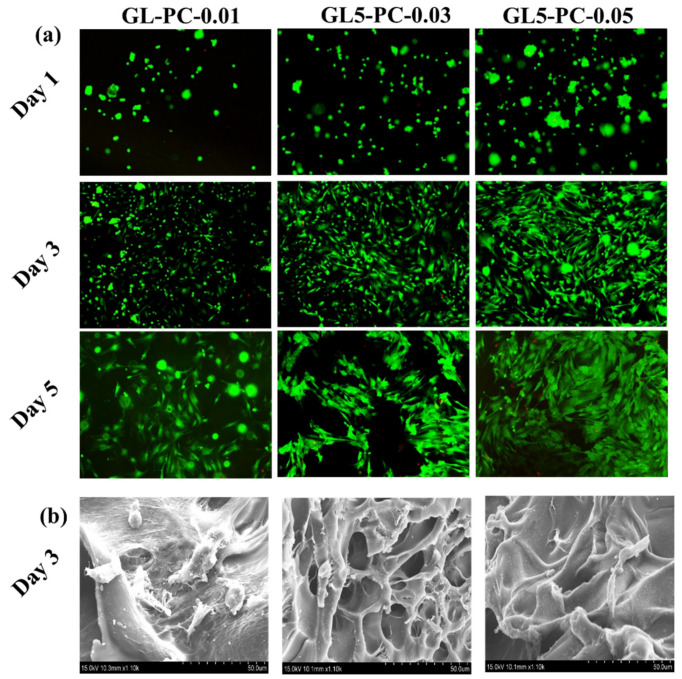
(**a**) Live/dead fluorescence images of BSCs (isolated from 30-month-old Korean male cattle) grown on GL-PC hydrogels (green = live cells staining with calcein-AM, red = dead cells staining with EthD-1) and (**b**) SEM images of BSCs cultured on GL-PC hydrogels after 3 days incubation.

We sought to assess the early cell–scaffold interactions necessary to produce cultured meat. For this purpose, a PrestoBlue test and live/dead studies were used to evaluate the cell viability of BSCs cultivated in GL-PC. We chose to conduct a direct culture for 1, 3, and 5 days to see if the cell viability results after 1 day of direct cell culture are the consequence of the cell–hydrogel interactions of the studied hydrogels themselves or due to the prolonged adaptation of the cells. The BSCs cultured on the hydrogels maintained 100% cell viability after 5 days of incubation (Figure 6). The PCs did not show any toxicity towards BSCs. The catechol groups on the PCs were responsible for the good growth of muscle cells on the GL hydrogels. The SEM images showed that the surface morphology changed with the increasing concentration of PCs in the GL hydrogels. Therefore, the morphology of the hydrogel surfaces may also be due to the maintenance of good cell viability at higher concentrations of PCs for 5 days of incubation. Further, live/dead staining was performed after the BSCs were cultured on GL-PC hydrogels for 1, 3, and 5 days (Figure 7a). Green fluorescent staining was observed for live cells, whereas red fluorescent staining was observed for dead cells. BSCs seeded on hydrogels showed aggregated cellular morphology after 1 day, whereas cells were spread on all hydrogels after 3 and 5 days of incubation. Furthermore, the spreading of cells was improved by increasing the PC amount in the hydrogels. A few dead cells were observed in the GL5PC-0.05 hydrogel after 5 days of incubation. However, these had a negligible effect on the cell viability of the GL-PC hydrogel. Therefore, the PCs could improve the cell spreading on the hydrogels. Overall, the addition of PCs did not hinder initial cell–biomaterial interactions and enhanced the cell spreading capability after 5 days of incubation. Figure 7b depicts the cell adhesion behavior of the GL-PC hydrogels after 3 days of culture. Cell proliferation can be clearly observed in the GL-PC hydrogels. Additionally, the cell growth on the GL-PC hydrogels showed cell spreading and also provided a transient indication of cell migration in the form of cell protrusion. In general, the GL hydrogel showed moderate cell adhesion of muscle cells. In our findings, the cell adhesion was greatly improved by increasing the amount of PCs in the hydrogels. The PC itself enhanced the growth of muscle cells similar to the enhancement seen with growth factors. Therefore, the GL-PC hydrogels have good features that encourage the growth and adhesion of muscle cells and can thus be potentially suitable for cultured meat applications.

## 3. Conclusions

In conclusion, a simple low-cost method has been employed for the preparation of GL-based hydrogels using PCs. The results of SEM and compressive studies revealed that the PCs can influence the morphological and compressive properties of the GL hydrogels. A preliminary cell study was performed using BSCs on GL-PC hydrogels. The results show that cell proliferation and adhesion of BSCs were enhanced by increasing the concentration of PCs in the hydrogels. To produce a product that resembles meat, muscle cells must be differentiated, and this needs to be investigated in future work.

## 4. Materials and Methods

### 4.1. Materials

GL (type A from porcine skin) was purchased from Sigma Aldrich, Saint Louis, USA. Food grade grape seed extract (95% proanthocyanidins (PCs)) powder was purchased from the NuSci Institute & Corp., Walnut, USA.

### 4.2. Fabrication of GL-PC Porous Hydrogels

GL (5% *w*/*v*) was prepared in double distilled water (DDW) at 50 °C. Then, the GL solution was poured into molds and freeze-dried. The freeze-dried GL hydrogels were immersed in various amounts of PCs in phosphate-buffered saline (PBS) solutions (0.01, 0.03, and 0.05% *w*/*v*). The resulting hydrogels were washed with PBS and DDW and then freeze-dried for further studies.

### 4.3. Characterization

The morphological features of the hydrogels were studied by scanning electron microscopy (SEM) at an accelerating voltage of 10 kV. Prior to imaging, the freeze-dried hydrogels were coated with platinum metal using an ion sputter for 60 sec. Compression testing of hydrogels was performed (MCT 2150, A&D Co., Ltd., Tokyo, Japan) using a cylindrical shape of hydrogel samples with dimensions of diameter 10 mm × length 10 mm with a 5 kN load at a speed of 50 mm/min^−1^.

### 4.4. Cell Culture

BSCs were sourced from local slaughterhouses and isolation was performed at the Chungbuk National University, Cheongju-si, South Korea, in a fresh state within four hours of slaughter. After the muscle tissue was disinfected, it was minced using a blade and dispersed in a collagenase dispersion. After 1 h of incubation, it was pipetted with an 18-gauge needle for physical separation. The ECM was sequentially filtered at 100, 70, and 40 micro meshes. BSCs were grown in Dulbecco’s Modified Eagle’s Medium (DMEM), which also contained 1% antibiotic-antimycotic (Gibco BRL) solution, 10 M p38i (Selleck, S1076), 5 ng/mL basic fibroblast growth factor (bFGF), and 20% fetal bovine serum (FBS). A 5% CO_2_, humidified environment at 37 °C was used to cultivate the cells.

The cell viability of the BSCs (5 × 10^4^ cells/cm^2^) on the sterilized porous hydrogels was evaluated using the PrestoBlue cell viability reagent (Thermo Fisher Scientific Korea Co., Ltd., Seoul, Republic of Korea) in a 48-well plate which was incubated for 1, 3, and 5 days. The media was removed, and 900 µL of fresh media was added with 100 µL PrestoBlue solution. The well plate was incubated at 37 °C for 30 min, and the solution was transferred to a 96-well plate. The solutions optical density was analyzed at 570 nm using a microplate reader.

Using a Live/Dead assay kit, the BSCs (5 × 10^4^ cells/cm^2^) were seeded on the hydrogel and cultured for 1, 3, and 5 days to test the cells’ biocompatibility and cell adhesion (Thermo Fisher Scientific Korea Co., Ltd., Seoul, Republic of Korea). The hydrogel was treated with 300 µL of Live/Dead solution (10 mL of PBS with 20 µL of ethidium homodimer-1 and 2 µL of calcein-AM). The hydrogel was then incubated at 37 °C for 30 min. Fluorescence microscopy was used to obtain the images (Live/Dead cells) (Nikon Eclipse Ti, Genova, Italy).

For cell adhesion, BSCs (5 × 10^4^ cells/cm^2^) were seeded on the hydrogel and incubated for 3 days. An amount of 500 µL of 2.5% glutaraldehyde was added to the cell-grown hydrogel for fixation at 4 °C for 30 min. The hydrogel was washed several times with PBS and treated with osmium tetroxide (OsO_4_) for post-fixation. After that, gradients of aqueous ethanol solution were used to dehydrate the hydrogel. SEM was used to examine the morphology of the BSCs on hydrogel (SEM; Hitachi S-4800, Tokyo, Japan).

## Data Availability

Not applicable.

## References

[1] Hubalek S., Post M.J., Moutsatsou P. (2022). Towards resource-efficient and cost-efficient cultured meat. Curr. Opin. Food Sci..

[2] Hong T.K., Shin D.M., Choi J., Do J.T., Han S.G. (2021). Current issues and technical advances in cultured meat production: A review. Food. Sci. Anim. Resour..

[3] Ng S., Kurisawa M. (2021). Integrating biomaterials and food biopolymers for cultured meat production. Acta Biomater..

[4] Bomkamp C., Skaalure S.C., Fernando G.F., Ben-Arye T., Swartz E.W., Specht E.A. (2022). Scaffolding biomaterials for 3D cultivated meat: Prospects and challenges. Adv. Sci..

[5] Celikkin N., Rinoldi C., Costantini M., Trombetta M., Rainer A., Święszkowski W. (2017). Naturally derived proteins and glycosaminoglycan scaffolds for tissue engineering applications. Mater. Sci. Eng. C.

[6] Yang L., Yaseen M., Zhao X., Coffey P., Pan F., Wang Y., Xu H., Webster J., Lu J.R. (2015). Gelatin modified ultrathin silk fibroin films for enhanced proliferation of cells. Biomed. Mater..

[7] Yi H., Forsythe S., He Y., Liu Q., Xiong G., Wei S., Li G., Atala A., Skardal A., Zhang Y. (2017). Tissue-specific extracellular matrix promotes myogenic differentiation of human muscle progenitor cells on gelatin and heparin conjugated alginate hydrogels. Acta Biomater..

[8] Sung H.W., Huang D.M., Chang W.H., Huang R.N., Hsu J.C. (1999). Evaluation of gelatin hydrogel crosslinked with various crosslinking agents as bioadhesives: In vitro study. J. Biomed. Mater. Res..

[9] Levi S., Yen F.C., Baruch L., Machluf M. (2022). Scaffolding technologies for the engineering of cultured meat: Towards a safe, sustainable, and scalable production. Trends. Food Sci. Technol..

[10] Saaty A.H. (2022). Grapefruit Seed Extracts’ Antibacterial and Antiviral Activity: Anti-Severe Acute Respiratory Syndrome Coronavirus 2 Impact. Arch. Pharm. Pract..

[11] Han B., Jaurequi J., Tang B.W., Nimni M.E. (2003). Proanthocyanidin: A natural crosslinking reagent for stabilizing collagen matrices. J. Biomed. Mater. Res. Part A Off. J. Soc. Biomater. Jpn. Soc. Biomater. Aust. Soc. Biomater. Korean Soc. Biomater..

[12] Pei X., Wang J., Cong Y., Fu J. (2021). Recent progress in polymer hydrogel bioadhesives. J. Polym. Sci..

[13] Chen E.J., Novakofski J., Jenkins W.K., O’Brien W.D. (1996). Young’s modulus measurements of soft tissues with application to elasticity imaging. IEEE Trans. Ultrason. Ferroelectr. Freq. Control.

[14] Padhi A., Nain A.S. (2020). ECM in differentiation: A review of matrix structure, composition and mechanical properties. Ann. Biomed. Eng..

[15] Suneetha M., Rao K.M., Han S.S. (2019). Mussel-inspired cell/tissue-adhesive, hemostatic hydrogels for tissue engineering applications. ACS Omega.

[16] He Y., Guo S., Chang R., Zhang D., Ren Y., Guan F., Yao M. (2022). Facile preparation of antibacterial hydrogel with multi-functions based on carboxymethyl chitosan and oligomeric procyanidin. RSC Adv..

